# Predictors of heart failure symptoms in hereditary hemorrhagic telangiectasia patients with hepatic arteriovenous malformations

**DOI:** 10.1186/s13023-021-02109-4

**Published:** 2021-11-18

**Authors:** Lucas R. Cusumano, Joseph A. Tesoriero, Craig B. Wilsen, James Sayre, Matthew Quirk, Justin P. McWilliams

**Affiliations:** 1 Division of Interventional Radiology, Department of Radiology, David Geffen School of Medicine at UCLA, 757 Westwood Plaza, 2nd Floor, Room 2125, Los Angeles, CA 90095 USA; 2 Department of Biostatistics, Fielding School of Public Health, University of California, Los Angeles, CA USA

**Keywords:** Hereditary Hemorrhagic Telangiectasia, Arteriovenous malformations, High-output heart failure, Common hepatic artery, Computed tomography

## Abstract

**Background:**

Hepatic arteriovenous malformations (AVMs) in hereditary hemorrhagic telangiectasia (HHT) patients are most commonly hepatic artery to hepatic venous shunts which can result in high-output heart failure. This condition can be debilitating and is a leading cause of liver transplantation in HHT patients. However, it is not known what characteristics can discriminate between asymptomatic patients and those who will develop heart failure symptoms.

**Results:**

176 patients with HHT were evaluated with computed tomography angiography (CTA) between April 2004 and February 2019 at our HHT Center of Excellence. 63/176 (35.8%) patients were found to have hepatic AVMs on CTA. 18 of these patients were excluded because of the presence of another condition which could confound evaluation of heart failure symptoms. In the remaining 45 patients included in our cohort, 25/45 (55.6%) patients were classified as asymptomatic and 20/45 (44.4%) were classified as symptomatic, and these groups were compared.

In symptomatic patients, mean common hepatic artery (CHA) diameter was significantly higher (11.1 versus 8.4 mm) and mean hemoglobin levels were significantly lower (10.7 vs 12.6 g/dL). A stepwise multiple logistic regression analysis demonstrated that both CHA diameter and hemoglobin level were independent predictors of heart failure symptoms with ORs of 2.554 (95% CI 1.372–4.754) and 0.489 (95% CI 0.299–0.799), respectively. The receiver operator characteristic (ROC) curve of our analysis demonstrated an AUC of 0.906 (95% CI 0.816–0.996), sensitivity 80.0% (95% CI 55.7–93.4%), and specificity 75.0% (95% CI 52.9–89.4%).

**Conclusions:**

CTA is an effective and easily reproducible method to evaluate hepatic involvement of HHT. Utilizing CTA, clinical, and laboratory data we determined CHA diameter and hemoglobin level were independent predictors of heart failure symptoms.

## Background

Hereditary hemorrhagic telangiectasia (HHT) is an autosomal dominant disorder characterized by the development of arteriovenous malformations (AVMs) in the skin, mucous membranes, brain and visceral organs [1]. Hepatic manifestations of HHT are characterized by abnormal shunts that connect the hepatic arterial, hepatic venous or portal venous systems [2–5]. Hepatic artery to hepatic vein shunts are the most common type, and may result in high-output cardiac failure secondary to low resistance and high flow [6, 7]. High-output heart failure can become debilitating and is a leading cause of liver transplantation in HHT patients [8–10]. However, symptoms are nonspecific, often beginning with exertional dyspnea which may then progress to worsening dyspnea at rest [6]. Specifically in HHT, symptomatic heart failure has been defined as shortness of the breath in the absence of other confounding factors such as anemia or clinically significant pulmonary arteriovenous malformations (PAVMs) [11].

Most patients with liver AVMs are asymptomatic [8]. A prospective cohort study evaluating HHT patients with hepatic AVMs estimated an 1.4 per 100 person-years incidence of high-output heart failure [10]. In the 14 patients with high-output heart failure included in that study, one patient required a liver transplantation and four patients had no response to treatment with progression to death [10]. However, it is not known what characteristics can discriminate between the minority who will develop heart failure and the majority who will not. Furthermore, it is not known if prophylactic treatment of patients with liver AVMs can prevent development of symptoms. Current guidelines recommend treatment for liver AVMs only in symptomatic patients [12].

Computed tomography angiography (CTA) has been proposed as the most effective non-invasive modality for diagnosing hepatic involvement and evaluating different intrahepatic vascular shunts in HHT patients [13]. CTA characteristics associated with the development of symptomatic heart failure have not previously been identified. The aim of this study is to describe the clinical and CTA characteristics of HHT patients with hepatic AVMs and their association with symptomatic heart failure.

## Methods and materials

### Patients

The protocol for this retrospective study has been approved by the institutional review board and waived informed consent for participation. Our database comprised patients with a clinical or genetic diagnosis of HHT and a CTA including the hepatic vasculature performed at our HHT Center of Excellence between April 2004 and February 2019. Patients with a definite clinical diagnosis of HHT had at least three of the following criteria present: epistaxis, telangiectasia, visceral lesions, or a first degree relative with HHT [4].

### Clinical characteristics

The presence of heart failure symptoms, defined as exercise-induced fatigue and dyspnea, were determined by review of the electronic medical record (EMR). Patients were classified as “asymptomatic” if there was no limit to physical activity and ordinary activity did not induce symptoms. Patients who exhibited exercise induced fatigue and dyspnea were considered “symptomatic,” and were classified using the New York Heart Association (NYHA) functional classification [14]. We excluded patients with other conditions which may produce symptoms that overlap with heart failure, such as untreated PAVMs, symptomatic anemia or asthma [15–20]. Patients were deemed to have symptomatic anemia and excluded from the study if symptoms correlated with a clinically significant drop in hemoglobin or bleeding event in which symptoms resolved following correction. We also used a threshold hemoglobin level of 7 g/dl to exclude patients because anemia related heart failure has been proposed to occur below this level [21].The presence of atrial fibrillation was determined from review of the EMR. Evidence of portal hypertension was assessed by the presence of splenomegaly, ascites, variceal bleeding, or encephalopathy [22].

### Computed tomography angiography

CTA of the chest or abdomen was performed on a Siemens 64-detector scanner (Siemens, Erlangen, Germany). Chest CTA was performed from one centimeter superior to the lung apices to the mid kidney level using a single breath hold and with the patient supine and their arms above the head. Abdominal CTA was performed from one centimeter superior to the diaphragm to the iliac crest. Iodine-based contrast material was administered through a peripheral vein at a flow rate of 4.0 mL/s for a total of 100 mL followed by 100 mL saline bolus administered at the same rate. Image acquisition was triggered for chest CTA when the main pulmonary artery reached 100 Hounsfield unit (HU) attenuation and for abdominal CTA when the abdominal aorta just above the diaphragm reached 150 HU attenuation. Timing of image acquisition allowed for sufficient opacification of hepatic vasculature.

CTAs were evaluated for the presence of hepatic AVMs, diameter of the common hepatic artery (CHA), diameter of the right and left hepatic arteries, portal vein diameter and main pulmonary artery diameter.

### Laboratory values

Laboratory values collected were the most recent with respect to CTA examination. To reflect routine laboratory values available for most HHT patients seen in the clinic setting, laboratory values included in this study were total bilirubin, albumin, platelet count, and hemoglobin. Additional laboratory values proposed to be helpful in the diagnosis of heart failure, such as natriuretic peptide, were not evaluated as they were only available in a minority of patients [23].

### Echocardiograms

For patients with echocardiograms available, the cardiac chamber size, ejection fraction, degree of valvular regurgitation and right ventricular systolic pressure were determined from the echocardiogram performed closest to date of the CTA. Chamber size of the right ventricle, left atrium, and left ventricle was graded on a scale from non-dilated to severely dilated by the interpreting cardiologist. Degree of valvular regurgitation of the tricuspid, mitral and aortic valves were graded on a scale from no regurgitation to severe regurgitation by the interpreting cardiologist.

### Statistical analysis

Continuous data are expressed as mean ± standard deviation. Comparisons between groups were made using the Fisher’s exact test for categorical variables and Mann–Whitney analysis for continuous variables. *P* values < 0.05 were considered significant. Stepwise multiple logistic regression analysis was performed to determine factors impacting patients experiencing heart failure symptomatology. ORs were reported with 95% CIs. Factors used included age, CHA diameter, evidence of portal hypertension, portal vein diameter, pulmonary artery diameter, platelet count, and hemoglobin. Parameters with the highest performance in the stepwise logistic regression analysis were evaluated using receiver operator characteristics (ROC). AUC, sensitivity, and specificity were calculated to determine the diagnostic value of these parameters in determining the presence of heart failure symptoms. Statistical analyses were performed with SPSS software, version 24.0 (IBM Corp, Armonk, New York) and GraphPad Prism, version 8.0 (GraphPad Software, La Jolla, California).

## Results

### Patient characteristics

**Fig. 1:**
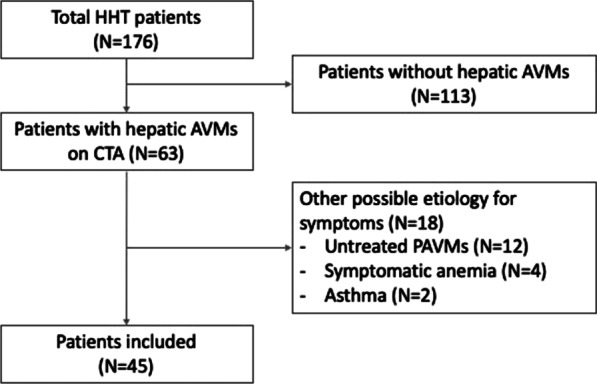
Flow chart of patient inclusion

At our HHT Center of Excellence, 521 patients with definite HHT were examined between April 2004 and February 2019. CTA examinations were available in 176 patients HHT patients.. Patient age ranged between 18 and 81 years old with a mean age of 49.5 (± 15.9) years. 107/176 (60.8%) were female. 63/176 (35.8%) patients were found to have hepatic AVMs on CTA. 18 of these patients were excluded because of the presence of another condition which could confound heart failure symptoms. As a result, our cohort included 45 patients (Fig. 1).

HHT was genetically confirmed in 30/45 (66.7%) patients; the remaining 15/45 (33.3%) patients met criteria for a clinical diagnosis of HHT [4]. 25/45 (55.6%) patients were classified as asymptomatic and 20/45 (44.4%) were classified as symptomatic. There was no significant difference in age, sex or presence of portal hypertension between groups. Symptomatic patients were more likely to have a history of atrial fibrillation (*P* = 0.0337) (Table 1).

**Table 1:** Clinical characteristics

Characteristic	All (N = 45)	Asymptomatic (N = 25)	Symptomatic (N = 20)	*P* value
Age (years)	57.2 (± 15.8)	53.7 (± 16.6)	61.5 (± 13.9)	0.1195
Sex (female)	33 (73.3%)	18 (72.0%)	15 (75.0%)	> 0.9999
Atrial fibrillation	7 (15.6%)	1 (4.0%)	6 (30.0%)	0.0337
Portal hypertension	2 (4.4%)	0 (0%)	2 (10.0%)	0.1058

### Computed tomography angiography

CHA diameter and right hepatic artery diameter were significantly larger in symptomatic patients. Left hepatic artery diameter was also larger in symptomatic patients but this difference did not reach statistical significance. Mean portal vein diameter was enlarged in both groups (> 13 mm), however there was no significant difference between groups. Pulmonary artery diameter was also not significantly different between groups (Table 2).

**Table 2:** Computed tomography angiography characteristics

Characteristic	All (N = 45)	Asymptomatic (N = 25)	Symptomatic (N = 20)	*P* value
Common hepatic artery diameter (mm)	9.6 (± 2.5)	8.4 (± 2.1)	11.1 (± 2.2)	0.0003
Left hepatic artery diameter (mm)	6.2 (± 2.2)	5.6 (± 2.1)	6.9 (± 2.1)	0.0882
Right hepatic artery diameter (mm)	7.5 (± 2.4)	6.4 (± 1.8)	8.8 (± 2.0)	0.0006
Portal vein diameter (mm)	14.6 (± 3.0)	14.2 (± 3.0)	15.1 (± 3.2)	0.5507
Pulmonary artery diameter (mm)	27.1 (± 4.9)	26.0 (± 4.7)	28.6 (± 4.7)	0.1014

### Laboratory values

Laboratory values were available for 42/45 (93.3%) patients. Albumin was significantly higher in asymptomatic patients (4.2 g/dL vs 3.9 g/dL, *P* = 0.0344). Hemoglobin was also significantly higher in asymptomatic patients (12.6 g/dL vs 10.7 g/dL*, P* = 0.0097). Total bilirubin and platelet count showed no significant difference between groups (Table 3).

**Table 3:** Laboratory values characteristics

Characteristic	All	Asymptomatic	Symptomatic	*P* value
Albumin (g/dL)	4.1 (± 0.5)	4.2 (± 0.4)	3.9 (± 0.4)	0.0344
Total bilirubin (g/dL)	0.6 (± 0.3)	0.5 (± 0.2)	0.6 (± 0.4)	0.6863
Platelets (10^3^/μL)	242.3 (± 89.1)	244.9 (± 69.8)	239.1 (± 109.7)	0.9535
Hemoglobin (g/dL)	11.7 (± 2.3)	12.6 (± 2.0)	10.7 (± 2.3)	0.0097

### Echocardiogram characteristics

Echocardiogram studies were available in for 32/45 (71.1%) patients, including 17/25 (68.0%) asymptomatic patients and 18/20 (90.0%) symptomatic patients. Two patients had incomplete examinations with evaluation of one chamber size or valve unavailable. Although chamber enlargement and valvular regurgitation were more frequent in the symptomatic group, there was no significant difference between groups in echocardiogram characteristics (Table 4).

**Table 4:** Echocardiogram characteristics

Characteristic	All	Asymptomatic (N = 17)	Symptomatic (N = 18)	*P* value
Right ventricle dilation	6 (19.4%)	1 (5.9%)	5 (27.8%)	0.1774
Left atrial dilation	21 (65.6%)	8 (47.1%)	13 (72.2%)	0.1756
Left ventricle dilation	7 (21.9%)	3 (17.6%)	4 (22.2%)	> 0.9999
Tricuspid regurgitation	15 (48.4%)	5 (29.4%)	10 (58.8%)^1^	0.1663
Mitral regurgitation	10 (31.3%)	3 (17.6%)	7 (38.9%)	0.2642
Aortic regurgitation	8 (25.0%)	3 (17.6%)	5 (27.8%)	0.6906
Ejection fraction	63.6 (± 4.7)	63.5 (± 3.9)	63.3 (± 5.3)	0.8907
Right ventricular systolic pressure	34.1 (± 18.1)	33.9 (± 15.0)	38.8 (± 17.9)	0.4854

^1^Echocardiogram of characteristic unavailable for one patient

### Predictors of heart failure symptoms

**Fig. 2:**
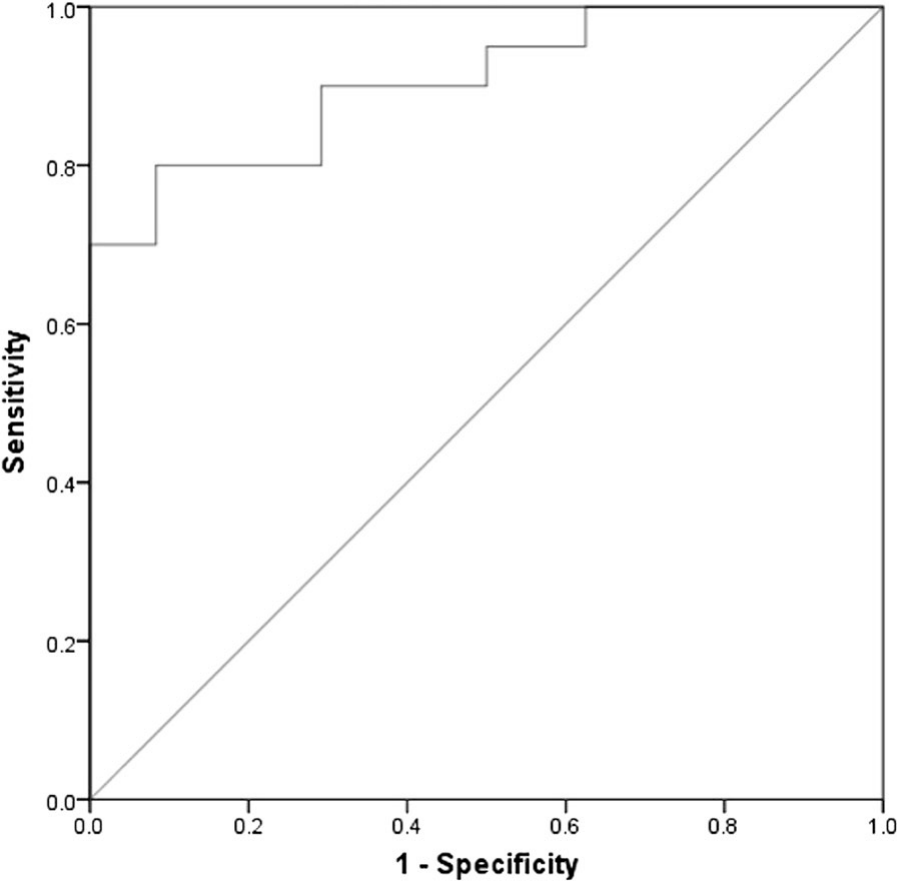
ROC curve of variables with the highest performance in the stepwise multivariate logistic regression analysis

In the stepwise logistic regression analysis, CHA diameter and hemoglobin were determined to be independent predictors of heart failure symptoms (Table 5). To assess the utility of these quantitative parameters as diagnostic tools, we performed ROC curve analysis. The ROC curve shows an AUC of 0.906 (95% CI 0.816–0.996), sensitivity 80.0% (95% CI 55.7–93.4%), and specificity 75.0% (95% CI 52.9–89.4%) (Fig. 2).

**Table 5:** Significant predictors of heart failure symptoms as determined by stepwise logistic regression analysis

Characteristic	OR	95% CI
Common hepatic artery diameter (mm)	2.554	1.372–4.754
Hemoglobin (g/dL)	0.489	0.299–0.799

## Discussion

Hepatic involvement of HHT is a complex process involving shunts between hepatic arterial, hepatic venous or portal venous systems [9, 13]. Hepatic AVMs may be present in up to 74% of HHT patients and several modalities have been utilized in their detection and monitoring [5]. Hepatic angiography is considered the gold standard to detect subtle abnormalities indicative of hepatic AVMs, however it is limited by the invasive nature of the procedure and has largely been replaced by non-invasive modalities such as ultrasonography and CTA [24]. While it is known that CTA can readily distinguish between different hepatic shunts and is often performed to characterize hepatic AVMs, no previous studies have quantified the association between CTA findings and the presence of heart failure symptoms in HHT patients [1, 5, 25].

The first international guidelines for HHT published in 2009 did not recommend routine screening of asymptomatic patients for hepatic AVMs, and recommended diagnostic imaging with Doppler ultrasound or CT for symptomatic patients [24]. In 2011, the publication of a large prospective cohort study showing high rates of hepatic AVM-related complications (25.3%) and death (5.2%) prompted our center to begin screening a high proportion of our adult HHT patients [10]. As technologist expertise in liver Doppler ultrasound for hepatic AVMs was lacking at our center, we chose CT angiography as our primary screening and diagnostic tool for liver AVMs. Using a chest CTA protocol with images extending through the liver, a single examination could be used to detect and characterize both pulmonary and hepatic AVMs. In symptomatic patients, dedicated CTA liver or dual-phase CT liver was performed. Computed tomography has the downside of radiation exposure, but has several advantages, including ready availability, rapid image acquisition, reproducibility, and lack of dependence on variable technologist expertise. Also, the ability to screen and diagnose AVMs with a single examination was useful for patients traveling long distances to reach our center and patients with uncertain follow-up. The second international HHT guidelines published in 2020 validated this approach, recommending that all adult HHT patients be offered hepatic AVM screening, using either Doppler ultrasound, or in centers where such expertise is lacking, contrast CT or magnetic resonance imaging (MRI) [12]. Identifying these hepatic AVMs and subsequently assessing their risk may lead to better outcomes even in asymptomatic HHT patients due to the early detection of hepatic AVM related complications [10, 24]. In concordance with international guidelines, we refer patients with symptomatic hepatic AVMs for echocardiography and evaluation by a cardiology specialist within our center [12].

The results of our multivariate analysis suggest that the degree of CHA dilation is an independent predictor of heart failure symptoms. Mean CHA diameter was significantly higher in symptomatic patients (11.1 versus 8.4 mm), and our data showed 2.6 times greater odds of having heart failure symptoms with each 1 mm increase in CHA diameter. These results are compatible with an ultrasonography study which demonstrated that high output heart failure only occurred in higher grade hepatic vascular malformations [10]. This ultrasound grading system is reproducible by trained ultrasonographers and incorporates factors such as dilation of the hepatic vasculature and vascular flow abnormalities [26]. Ultrasonography benefits by being low cost with a high sensitivity and specificity for liver AVM detection [10, 26–28]. However, it requires capable and experienced ultrasonographers, and the subjectivity of the grading system may lead to discrepancies between operators. On the other hand, measurement of the CHA diameter using CTA is unambiguous and is not technologist dependent.

Symptomatic anemia overlaps with heart failure symptoms, and hemoglobin less than 7 g/dL may directly lead to heart failure in a mechanism that expands extracellular plasma volumes from increased sympathetic and renin-angiotensin activity [29, 30]. Symptoms are temporary as correction of anemia demonstrates rapid and complete regression of anemia-related high output heart failure [29]. We excluded patients who experienced symptoms correlating to their anemia because of the possible overlap with heart failure symptoms. None of the patients in our cohort had hemoglobin levels less than 7 g/dL at the time of testing. Nevertheless, mean hemoglobin levels were significantly lower in symptomatic patients (10.7 vs 12.6 g/dL). Our multiple logistic regression analysis determined hemoglobin to be an independent predictor of heart failure symptoms with an OR 0.489 for each 1 g/dL increase of hemoglobin.

In our stepwise logistic regression analysis the combination of these two independent variables, CHA diameter and hemoglobin level, allows for excellent discrimination between patients who do and do not have heart failure symptoms with an AUC of 0.906. Employing the ORs respective to both CHA diameter and hemoglobin may help clinicians quantify risks to their patients.

Most patients in our cohort exhibited normal ejection fractions and a non-dilated left ventricle. In the remaining cardiac chambers, dilation was more frequently seen in the symptomatic group, though these differences were not statistically significant. Left atrial enlargement, for example, occurred in 72.2% of symptomatic patients, versus 47.1% of asymptomatic patients. The relatively small sample size likely contributed to this difference not reaching statistical significance and left atrial enlargement may be prognostic in a larger sample size. Additionally, the degree of regurgitation was higher in symptomatic patients but this also did not reach statistical significance. Although further research is needed, echocardiograms should continue to be utilized to monitor cardiac manifestations of HHT [31, 32].

Initial treatment of high-output heart failure secondary to liver AVMs is supportive and includes diuretics, salt and fluid restriction, beta-blockers and maintaining adequate hemoglobin levels [1, 22]. Bevacizumab, an anti-angiogenic agent, has been successfully used to reduce shunting and mitigate symptoms of high-output heart failure, but may expose patients to adverse events including hypertension and arterial thromboembolism [33]. Endovascular embolization is infrequently performed because of high complication risk from ischemic cholangitis, ischemic cholecystitis or hepatic necrosis [24]. Orthotopic liver transplant has been proposed as the only definitive curative treatment, but is typically reserved for patients with severe complications related to hepatic AVMs [8, 24, 34]. The current study shows only association, not causation, but measurement of CHA diameter and hemoglobin may help identify patients who are at higher risk of development of heart failure symptoms. This group may benefit from more aggressive correction of anemia, more frequent monitoring of symptoms and regular echocardiography. Further research is needed to explore if early or prophylactic treatment of this higher-risk group could improve outcomes associated with hepatic AVMs.

Our study had limitations inherent to studies with a small sample size performed at a single academic center and a retrospective design. Although we determined CHA and hemoglobin levels were independent predictors of heart failure symptoms, our cohort was not sufficiently powered to correlate these predictors with the degree of heart failure symptoms. Patients at our institution were not universally screened for hepatic AVMs over the study period and abdominal CTAs were often performed in patients with suspected hepatic AVMs due to symptoms. This likely led to the high proportion of symptomatic patients in our cohort. In our study, 44.4% of patients with hepatic AVMs were classified as having symptoms attributed to hepatic AVMs compared to the 8–15% of patients reported in the literature [5, 10, 24, 26]. Although chest CTAs were typically performed to screen for or evaluate the presence of PAVMs, they were included in this study because our chest CTA protocol includes the liver parenchyma and the hepatic vasculature. It is possible that hepatic AVMs in the inferior liver could have been excluded from the scan plane in some patients, causing mis-classification. These two factors could have led to exclusion of some patients with small, asymptomatic hepatic AVMs which would have only been detected by more extensive screening. Lastly, echocardiograms were not available in two symptomatic patients which limits the ability to exclude other cardiac causes for their heart failure symptoms.

## Conclusion

CTA is an effective and easily reproducible method to evaluate hepatic involvement of HHT. Combining CTA, clinical and laboratory data we found that common hepatic artery diameter and hemoglobin level were significantly associated with heart failure symptoms.

## Data Availability

The datasets generated and/or analysed during the current study are not publicly available due concerns for patient privacy but are available from the corresponding author on reasonable request.

## Abbreviations

AVM: Arteriovenous malformation
CHA: Common hepatic artery
CTA: Computed tomography angiography
EMR: Electronic medical record
HHT: Hereditary hemorrhagic telangiectasia
HU: Hounsfield unit
NYHA: New York Heart Association
PAVM: Pulmonary arteriovenous malformations
ROC: Receiver operator characteristic
AUC: Area under curve
MRI: Magnetic resonance imaging
